# Parental influences on adolescents' physical activity and sedentary behavior: longitudinal findings from Project EAT-II

**DOI:** 10.1186/1479-5868-5-12

**Published:** 2008-02-26

**Authors:** Katherine W Bauer, Melissa C Nelson, Kerri N Boutelle, Dianne Neumark-Sztainer

**Affiliations:** 1 Division of Epidemiology & Community Health, School of Public Health, University of Minnesota, Minneapolis, Minnesota, USA; 2 Division of General Pediatrics and Adolescent Health, Department of Pediatrics University of Minnesota, Minneapolis, Minnesota, USA; 3 Department of Pediatrics and Psychiatry, University of California San Diego, San Diego, California, USA

## Abstract

**Background:**

The long-term role that parental encouragement and attitudes about fitness and exercise play in adolescents' physical activity and sedentary behavior habits remains unclear. This paper aims to longitudinally examine how parental encouragement to be physically active and parental concern about staying fit are associated with adolescents' physical activity and sedentary behavior habits five years later.

**Methods:**

Project EAT-II adolescent and young adult participants (1130 male, 1386 female) completed surveys while in middle school or high school (1998–1999), and again 5 years later. Participants were asked whether their mother and father encourage them to be physically active and care about staying fit and exercising. Adolescent moderate and vigorous physical activity (MVPA) and TV/video watching (hours/week) were assessed. Linear regression models adjusted for socio-demographic characteristics and baseline behavior were used to examine the association of Time 1 parental factors with behavioral outcomes among adolescents and young adults five years later (Time 2).

**Results:**

After adjustment for socio-demographic characteristics and baseline MVPA, adolescent-reported maternal and paternal encouragement to be active, and paternal care for fitness, were positively associated with weekly hours of MVPA after five years in young adult males (p for trend ≤ .01). The positive relationship between maternal encouragement and MVPA approached significance among high-school aged females (p for trend = .06), and paternal encouragement was positively related to MVPA among high-school aged males (p for trend = .02). While maternal encouragement to be active was associated with decreased TV/video time among younger females (p for trend = .02), other parental factors were not associated with lower TV/video time among the other groups.

**Conclusion:**

Parental encouragement to be active was associated with increased physical activity among males and younger females 5 years later. Younger adolescents appear to be especially influenced by their same-sex parent. These findings suggest that encouragement may be more influential than parental concern for fitness on adolescents' physical activity habits. Further research is needed to determine how parents can help adolescents decrease sedentary behavior time.

## Introduction

The low level of physical activity among many adolescents is of public health concern. Studies performed in the United States have found that more than half of adolescents participate in moderate levels of physically activity less than 3 days a week[1] and on average engage in sedentary behaviors such as television watching for approximately 5 hours per day[2]. Additionally, physical activity levels tend to decrease as adolescents move into young adulthood[3], leaving them at risk for the poor health outcomes associated with physical inactivity, which include overweight and obesity [4-6]. Many researchers have suggested that parents play a critical role in adolescents' physical activity and sedentary behavior habits[7,8].

Several studies have utilized cross-sectional data to examine the relationship between parental support for and modeling of physical activity and adolescents' physical activity levels. The majority of these studies have found a positive relationship between parental encouragement to be physically active and adolescents' time spent engaging in physical activity and participation in organized sports [8-12]. One recent longitudinal study examining the relationship between family support and physical activity among adolescent girls confirms this positive relationship[13]. There is also some evidence that parental modeling of physical activity and concern for fitness is associated with greater levels of physical activity among adolescents, although that relationship is less consistent[11,14]. Fewer studies have explained the relationship between parental encouragement and attitudes about fitness and the time adolescents spend engaging in sedentary behaviors. These studies have produced mixed results [2,15].

Much of the previous research in this area has been cross-sectional, therefore additional longitudinal research is needed to determine the temporal relationship between parental factors and adolescents' activity habits. Longitudinal studies also enable us to assess the long-term relationship between parental factors and adolescents' physical and sedentary activities.

Additionally, little work to date has examined parent-specific (e.g. maternal vs. paternal) influences on adolescent males and females; it is possible that parents yield a gender-specific influence which is masked when maternal and paternal data are pooled. By addressing these issues and examining these relationships using the Project EAT-II cohort we can begin to gain insight into the temporal relationship between parental factors and adolescents' activity habits, and the long-term strength of that relationship. Thus, this paper aims to longitudinally examine the relationship between adolescent-reported parent-specific encouragement to be physically active and parental concern about staying fit, and adolescents' physical activity and sedentary behavior habits five years later.

## Methods

### Study design and population

Project EAT-II is a longitudinal, follow-up study of Project EAT-I, which explored the socioenvironmental, personal, and behavioral determinants of dietary intake and weight-related variables among an ethnically diverse adolescent population. Project EAT-I enrolled 4746 participants including a younger cohort of middle-school students and an older cohort of high-school students recruited during the 1998–1999 academic year from 31 middle and high schools with ethnically and racially diverse student populations. Project EAT-II aimed to resurvey the original participants by mail to assess changes in their eating patterns and weight status 5 years later (2003–2004). Of the original study population, 22.5% (1074) were lost to follow-up for various reasons, primarily missing contact information at Project EAT-I (n = 411) and no address found at follow-up (n = 591). Of the remaining 3672 participants who were contacted by mail, 2516 competed surveys. These 2516 participants represent 53.0% of the original study sample and 68.4% of participants who could be contacted for Project EAT-II. One-third of the participants (32.0%) were in the younger cohort; at Time 1, their mean age was 12.8 years (SD = 0.8) and at Time 2 their mean age was 17.2 years (SD = 0.6). Two-thirds of the participants (68.0%) were in the older cohort; at Time 1, their mean age was 15.8 years (SD = 0.8) and at Time 2 their mean age was 20.4 years (SD = 0.8). Although the younger cohort is smaller than the older, there is sufficient power to precisely estimate the relationships between the parental variables and adolescents' outcomes in both cohorts.

### Data collection

Surveys were sent by mail to the address provided by the participant during Project EAT-I. Methods to locate Project EAT-I participants and increase the survey response rate have been described elsewhere[3]. Data collection occurred between April 2003 and June 2004 and was conducted by the Data Collection and Support Services in the Division of Epidemiology and Community Health at the University of Minnesota. The University of Minnesota's Institutional Review Board Human Subjects Committee approved all study protocols.

### Measures

#### Physical activity and sedentary behavior

Project EAT-I and EAT-II surveys included several questions to assess physical activity and sedentary behavior. Questions related to physical activity were modified from the Leisure Time Exercise Questionnaire[16,17]. Two survey items individually assessed moderate and vigorous activity asking, "In a usual week, how many hours do you spend doing the following activities...." Vigorous activity was described as strenuous, during which the heart beats rapidly, and moderate activity was described as not exhausting. More than 10 examples of specific activities were given after each question. Possible responses ranged from 0 to ≥6 hours per week. A continuous variable representing total weekly hours of moderate and vigorous physical activity (MVPA) per week was created by summing the weekly hours of moderate and vigorous activity.

Items to measure time spent watching television and videos were adapted from Planet Health[18]. Participants reported average hours per weekday and weekend day (Saturday or Sunday) "watching TV & videos." Possible categorical responses ranged from 0 to ≥5 hours per day. These items have been validated and show moderate to high test-retest correlations. A continuous variable, total hours of TV & videos per week, was created by summing the average weekday hours multiplied by five, and average weekend hours, multiplied by two.

#### Adolescents' perception of parents

The Project EAT-I survey included two items asking how much the participant's mother encouraged them to be physically active and how much she cared about staying fit and exercising. The same two items asked about the participant's father's encouragement and care about staying fit and exercising. Possible response to these questions were: 1 = "not at all," 2 = "a little bit," 3 = somewhat," and 4 = "very much." These items had test-retest correlations between 0.66 and 0.69.

#### Sociodemographic characteristics

Gender, age, ethnicity/race and socioeconomic status (SES) were based on adolescents' self-report in Project EAT-I. The primary determinant of SES was parental educational level, defined by the highest level of educational attainment of either parent. In addition, an algorithm was developed to take into account family eligibility for public assistance, eligibility for free or reduced-cost school meals, and employment status of the mother and the father[19].

### Data analysis

Because attrition in the study population during the 5-year study did not occur completely at random, the data were weighted to adjust for differential response rates in Project EAT-II using a response propensity method[20]. The use of this method with Project EAT data has been described in detail elsewhere, where it has been evaluated as a means of correcting potential response bias[21]. At Time 1, non-responders to Project EAT-II reported participating in fewer MVPA hours per week (7.1 vs. 7.5, p < .01) and more TV/video hours per week (18.9 vs. 17.8, p < .01), compared to those who did participate in Project EAT-II. Additionally, there were some differences in the amount of parental encouragement and care for fitness reported by these two groups. After adjusting for sociodemographics and weighting, there were no significant differences found in MVPA nor TV/video hours per week at Time 1 between responders and nonresponders at Time 2. The weighted ethnic/racial and SES proportion are 48.5% white, 19.0% black, 19.2% Asian, 5.8% Hispanic, 3.5% Native American, and 3.9% mixed or other race. Thirty-seven percent of the sample were of low or low-middle SES.

ANOVA models were used to calculate cohort and gender-specific weighted means and standard errors of baseline parental factors, as measured during Project EAT-I (Time 1), and cohort and gender-specific weighted means and standard errors of the primary outcomes, hours of physical activity and sedentary behavior per week, as measuring during Project EAT-II (Time 2). The gender by cohort interaction was evaluated to determine if the pattern of means by gender of the parental variables or primary outcomes differed by cohort. If significant, post-hoc t-tests were used to test differences between the individual means.

Separate multiple linear regression models were developed to examine the association between maternal and paternal variables and the adolescents' behavioral outcomes of weekly hours of MVPA and TV/video watching five years later. All analyses were stratified by gender and cohort ("younger cohort" refers to those transitioning from junior high/middle school to high school and "older cohort" refers to those transitioning from high school to young adulthood) to assess the differential impact of mothers and fathers on children of the same or opposite sex, and potential differences in the relationship between parental influence and behavioral outcomes for the younger versus older cohort. All of the models were first adjusted for race and SES (Model 1), and then adjusted for baseline values of the primary outcome (i.e. adolescent physical activity or TV/video viewing) along with race and SES (Model 2).

## Results

Descriptive statistics for key variables at Time 1 and 2 are presented in Table 1. At Time 2, participants reported engaging in MVPA between 5.8 and 7.6 hours per week and reported watching TV/video between 17.1 and 19.2 hours per week. Older females report less parental caring about fitness at Time 1 as compared to younger females and males of both age groups. There were no other significant differences in perceived parental factors or activity habits by age or gender groups.

**Table 1:** Adolescent reports of parental influence variables at Time 1 and adolescent physical activity and sedentary behaviors at Time 2 by gender and cohort: Descriptive statistics

	**Younger Males^#^**		**Older Males^#^**		**Younger Females^#^**		**Older Females^#^**		Gender * Cohort Interaction	
	**N**	**Mean (SE)**	**N**	**Mean (SE)**	**N**	**Mean (SE)**	**N**	**Mean (SE)**	**F**	**p**

Maternal Influence Variables* (Time 1)										
Encouragement to be Active	347	3.13 (.05)	750	3.09 (.04)	417	3.07 (.05)	921	2.90 (.03)	2.25	.13
Cares About Staying Fit	348	2.95 (.05) ^a^	751	2.90 (.03) ^a^	417	2.99 (.05) ^a^	925	2.76 (.03) ^b^	4.64	.03
Paternal Influence Variables* (Time 1)										
Encouragement to be Active	339	3.07 (.06)	727	2.93 (.04)	382	2.90 (.06)	891	2.65 (.04)	1.12	.29
Cares About Staying Fit	339	2.89 (.06)	722	2.66 (.04)	383	2.76 (.05)	890	2.57 (.04)	.13	.72
**Primary Outcomes (Time 2)**										
Hours of MVPA per week	356	7.61 (.14)	749	7.36 (.09)	429	6.34 (.13)	936	5.79 (.08)	1.77	.18
Hours of TV & Video per week	357	19.08 (.54)	748	19.17 (.37)	427	17.14 (.50)	933	17.28 (.33)	0.00	.96

^#^Younger refers to participants who were in middle school at Time 1, and high school at Time 2, Older refers to participants who were in high school at Time 1, and were young adults at Time 2

* Scale = 1 – 4 (1 = not at all; 2 = a little; 3 = somewhat; 4 = very much)

Note: For each comparison, means with different letters were significantly different (p < .05), while means sharing the same letter were indistinguishable.

### Physical activity

Table 2 contains the results from the separate regression analyses examining the association between the adolescent-reported parent-specific factors at Time 1 and hours of physical activity per week at Time 2. Based on Model 1, in which the regression analyses are adjusted for race and SES, among younger and older males and younger females encouragement to be physically active by a same-sex parent was associated with greater hours of MVPA five years later (p for trend = <.01). For example, younger males who reported at Time 1 that their father 'very much' encourages them to be physically active engaged in MVPA 1.4 more hours per week at Time 2, compared to those that reported their father did not encourage them to be physically active. Maternal encouragement also predicted more hours of MVPA after 5 years among older males, with those reporting no encouragement participating in 6.6 hours of MVPA per week compared to 7.8 hours per week among those who reported that their mother "very much" encouraged them to be active (p for trend < .01).

**Table 2:** Adolescent physical activity at Time 2 by adolescent-reported parental factors at Time 1: 5-year longitudinal associations

		**n^a^**	**Model 1^b ^Adjusted Mean Hours of MVPA per Week by Level of Parental Factors**				**Model 1^b ^p for trend**	**Model 2^c ^p for trend**
		
			Not at all	A little	Somewhat	Very much		
Maternal Encouragement to be Active								
	Younger Males	324	7.3	7.6	7.7	7.9	0.22	0.96
	Older Males	731	6.6	6.9	7.2	7.8	<0.01	<0.01
	Younger Females	395	5.5	6.0	6.3	6.7	<0.01	0.06
	Older Females	907	5.6	5.7	5.9	5.9	0.30	0.68
Mother Cares About Staying Fit								
	Younger Males	325	7.4	7.2	8.2	7.6	0.31	0.93
	Older Males	732	7.3	7.1	7.6	7.3	0.60	0.97
	Younger Females	396	5.8	6.4	6.2	6.7	0.12	0.99
	Older Females	911	5.6	5.7	5.8	6.1	0.12	0.38

Paternal Encouragement to be Active								
	Younger Males	317	6.8	7.0	7.7	8.2	<0.01	0.02
	Older Males	708	6.7	7.0	7.7	7.7	<0.01	<0.01
	Younger Females	361	6.1	6.4	6.4	6.6	0.20	0.87
	Older Females	877	5.5	6.0	5.7	6.1	0.06	0.89
Father Cares About Staying Fit								
	Younger Males	317	6.3	7.3	8.0	8.1	<0.01	0.10
	Older Males	703	6.8	7.2	7.7	7.7	<0.01	0.01
	Younger Females	362	5.8	6.7	6.4	6.6	0.10	0.23
	Older Females	876	5.5	6.0	5.9	6.0	0.05	0.30

^a ^n may differ slightly across variables and models

^b ^Model 1: Adjusted for race (white/non white) and SES in 5 categories

^c ^Model 2: Adjusted for race (white/non white, SES in 5 categories and hours of MVPA at Time 1)

Adolescents' perception that their mother cares about staying fit and exercising was not related to hours of MVPA after 5 years. In contrast, paternal care for staying fit and exercising was related to increased MVPA hours among younger and older males (p for trend < .01) and among older females (p for trend = .05), in analyses adjusted for race and SES (Model 1).

After additional adjustment for baseline hours of MVPA (Model 2), the positive relationships between maternal and paternal encouragement, and paternal care for fitness, and MVPA among older males remained (p for trend ≤ .01). Additionally, the positive relationship between maternal encouragement and hours of MVPA among younger girls approached statistical significance (p for trend = .06), and the positive relationship between paternal encouragement and MVPA in younger males remained significant (p for trend = .02).

### Sedentary behavior

Adolescent-reported parental factors were less predictive of TV/video watching after five years (Table 3). After adjustment for race and SES (Model 1), younger females' perception that their mother encourages them to be active was related to lower levels of TV/video five years later (p for trend < .01); however maternal encouragement to be physically active was associated with higher levels of TV/video in older males (p for trend = .02). Younger females' perception that their mother cares about staying fit and exercising was also associated with lower levels of TV/video hours after five years (p for trend = .04). Paternal factors did not have any relationship to hours of TV/video watching per week among any of the groups.

**Table 3:** Adolescent sedentary activity at Time 2 by adolescent-reported parental factors at Time 1: 5-year longitudinal associations

		**n^a^**	**Model 1^b ^Adjusted Mean Hours of TV/Video per Week by Level of Parental Factors**				**Model 1 p for trend^b^**	**Model 2 p for trend^c^**
		
			Not at all	A little	Somewhat	Very much		
Maternal Encouragement to be Active								
	Younger Males	324	19.4	18.4	20.4	17.4	0.50	0.63
	Older Males	730	16.2	18.2	19.6	19.4	0.02	<0.01
	Young Females	393	21.7	19.7	16.7	15.9	<0.01	0.02
	Older Females	904	17.6	17.1	17.4	17.7	0.85	0.58
Mother Cares About Staying Fit								
	Young Males	325	18.4	19.7	18.7	18.0	0.78	0.94
	Older Males	731	19.1	19.1	18.4	19.9	0.72	0.30
	Young Females	394	20.9	17.0	17.6	16.3	0.04	0.45
	Older Females	908	16.9	17.9	17.5	17.2	0.94	0.92

Paternal Encouragement to be Active								
	Younger Males	317	17.9	20.6	19.8	17.4	0.72	0.24
	Older Males	707	18.4	18.8	18.3	19.4	0.46	0.04
	Young Females	359	18.3	17.1	18.0	14.9	0.08	0.66
	Older Females	874	18.7	16.4	17.7	17.2	0.29	0.22
Father Cares About Staying Fit								
	Young Males	317	17.3	18.8	19.3	18.1	0.67	0.15
	Older Males	702	19.2	19.4	18.0	19.3	0.78	0.20
	Young Females	360	17.7	15.9	16.9	16.5	0.61	0.27
	Older Females	873	18.4	17.1	17.2	17.5	0.40	0.32

^a ^n may differ slightly across variables and models

^b ^Model 1: Adjusted for race (white/non white) and SES in 5 categories

^c ^Model 2: Adjusted for race (white/non white, SES in 5 categories and hours of TV/video at Time 1)

After additional adjustment for baseline hours of TV/video watching (Model 2), the negative relationship between maternal encouragement to be physically active and TV/video use among younger females (p for trend = .02) remained, along with the positive relationship between maternal encouragement and TV/video use among older males (p for trend < .01). Additionally, a positive relationship between fathers' encouragement to be physically active and older males' hours of TV/video emerged (p for trend = .04).

## Discussion

The purpose of this study was to longitudinally examine how parental encouragement to be physically active and parental concern about staying fit are associated with adolescents' MVPA and TV and video watching behaviors after five years. Males' MVPA hours were predicted by paternal encouragement and care for fitness while younger females' MVPA was positively predicted by encouragement by their mothers, in analyses adjusting for sociodemographics. After additional adjustment for baseline levels of MVPA many of these relationships remained or approached statistical significance. These findings suggest the long-term importance of encouragement to be active by adolescents' same-sex parent.

The results of this study indicate that parental encouragement to be physically active can predict adolescents' physical activity habits, supporting previous evidence from the cross-sectional literature [8-12], including the review of correlates of physical activity among adolescents conducted by Sallis, et al. [8], and providing additional evidence to support Dowda, et al.'s recent findings that these may be lasting, long-term effects[13]. By examining the gender and cohort groups separately, we were able to determine that males and females may not be influenced equally by both of their parents. Additional research is needed to understand why these patterns of influence emerged and how to intervene to improve adolescents' physical activity habits.

With the exception of older males, parental concern about their own fitness was not associated with adolescents' MVPA habits after adjustment for baseline MVPA. This finding is similar to findings of Trost, et al[11] in suggesting that parents should actively encourage their children to be physically active, and not assume that parental attitudes about their own fitness, or modeling of fitness, affects the adolescent. Additionally, none of the parental factors examined predicted the MVPA habits of girls moving through high school and into young adulthood after adjustment for baseline MVPA. One possible reason for this lack of association is that girls of this age experience many competing influences that may affect their physical activity time. These influences include lack of support from peers or a school or neighborhood environment that doesn't encourage physical activity among high school-aged girls[22,23]. Further research is needed to determine how families can support physical activity among older adolescent girls.

Parental factors had a less consistent relationship with TV/video hours after five years. After adjustment for baseline TV/video, race, and SES, there is an unexpected positive relationship between parental encouragement to be physically active and TV/video use among older males, while a negative relationship is seen among the younger females. These mixed results align with what has been reported previously. These findings suggest that in order to reduce sedentary behavior among adolescents parents should not rely on general encouragement to be active or personal concern for fitness[2,10]. As physical activity and sedentary behavior have not been found to be strongly inversely related [6,24], it seems reasonable that different strategies work to encourage physical activity versus those that would decrease sedentary behavior. Additional longitudinal research is needed to determine which elements of the family environment are related to decreased sedentary behavior over time.

A strength of the current study was that the longitudinal design of Project EAT-II enabled us to examine associations between parent-specific factors and activity levels of adolescents over time. Additionally, the large and diverse nature of the study population allows for a fair amount of generalizability to other youth populations. However, in interpreting the findings, it is important to note that that this study only addressed two parental factors – encouragement and parental care for fitness. There may be other behaviors, such as providing logistical support to engage in physical activity or parents participating in physical activity with their child, that influence physical activity through adolescence and into young adulthood. Furthermore, it is noteworthy that the focus of the current analysis was on the association between adolescents' perceptions of their parents encouragement to be physically active and their personal concerns about staying fit. Research has suggested that adolescent perceptions of parental attributes may be a stronger determinant of adolescents' behaviors than parental reports of their own attitudes and behaviors [25].

## Conclusion

Building on previous research in this area, this research suggests that parental characteristics may influence adolescents' activity and sedentary behavior, though these relationships are highly complex. Our findings suggest that parental encouragement to be physically active is associated with increases in physical activity among some adolescents, and that encouragement to be active may be especially influential when coming from a same-sex parent. These findings have implications for family-based interventions aiming to increase adolescents' physical activity. This study suggests that in order to increase young adolescents' physical activity levels, and perhaps establish lifelong healthy habits, parents should actively encourage their children to participate in physical activity. Public health messages should also inform parents that modeling of physical activity or concern for fitness may not be enough to motivate their child, and physical activity-specific encouragement may be necessary. Additional longitudinal and intervention research is needed to find effective ways for parents to help reduce sedentary behaviors among their children.

## Competing interests

The author(s) declare that they have no competing interests.

## Authors' contributions

KWB, MCN, KNB and DNS contributed to the study design, KWB conducted the statistical analysis and wrote the manuscript, and all authors edited the manuscript and read and approved the final manuscript. DNS is the Principal Investigator of Project EAT-II.
