# Parental influences on adolescents' physical activity and sedentary behavior: longitudinal findings from Project EAT-II

**DOI:** 10.1186/1479-5868-8-12

**Published:** 2011-02-21

**Authors:** Katherine W Bauer, Melissa C Nelson, Kerri N Boutelle, Dianne Neumark-Sztainer

**Affiliations:** 1 Division of Epidemiology & Community Health, School of Public Health, University of Minnesota, Minneapolis, Minnesota, USA; 2 Division of General Pediatrics and Adolescent Health, Department of Pediatrics University of Minnesota, Minneapolis, Minnesota, USA; 3 Department of Pediatrics and Psychiatry, University of California San Diego, San Diego, California, USA

## 

Since publication of our article [1], we observed an error in the coding of the moderate and vigorous physical activity (MVPA) variable. This error affects the mean level of MVPA in the sample, but does not significantly change any of the associations observed between parentalbehaviors and adolescents' physical activity. Please find below a description of the amendments. Sentence numbers are counted from the beginning of the section/subsection. Amended version of tables 1 and 2 are also presented here.

**Table 1:** Adolescent reports of parental influence variables at Time 1 and adolescent physical activity and sedentary behaviors at Time 2 by gender and cohort: Descriptive statistics

Hours of MVPA per week row should read:										
Hours of MVPA per week	356	6.82 (.23)	749	6.43 (.16)	429	4.65 (.21)	936	3.86 (.14)	1.15	.24

**Table 2:** Adolescents physical activity at Time 2 by adolescent-reported parental factors at Time 1: 5-year longitudinal associations

		n^a^	Model 1^b ^Adjusted Mean Hours of MVPA per Week by Level of Parental Factors				Model 1^b^ p for trend	Model 2^c^ p for trend
			Not at all	A little	Somewhat	Very much		
Maternal Encouragement to be Active								
	Younger Males	324	6.4	7.0	6.9	7.3	0.35	0.79
	Older Males	731	5.1	5.7	6.1	7.3	< 0.01	< 0.01
	Younger Females	395	3.6	4.0	4.5	5.2	0.02	0.06
	Older Females	907	4.0	3.9	4.0	3.9	0.79	0.17
Mother Cares About Staying Fit								
	Younger Males	325	6.2	6.4	7.8	6.8	0.30	0.97
	Older Males	732	6.1	5.9	6.9	6.4	0.42	0.77
	Younger Females	396	3.6	4.6	4.5	5.2	0.06	0.55
	Older Females	911	3.9	3.8	3.8	4.2	0.46	0.77
								
Paternal Encouragement to be Active								
	Younger Males	317	5.7	5.8	6.8	7.9	< 0.01	0.05
	Older Males	708	5.4	5.6	6.9	7.0	< 0.01	< 0.01
	Younger Females	361	4.2	4.6	4.8	5.1	0.23	0.68
	Older Females	877	3.7	4.2	3.6	4.2	0.40	0.57
Father Cares About Staying Fit								
	Younger Males	317	4.9	6.3	7.4	7.6	< 0.01	0.19
	Older Males	703	5.7	6.0	6.9	7.0	0.01	0.02
	Younger Females	362	3.8	5.0	4.9	4.9	0.11	0.12
	Older Females	876	3.6	4.1	4.0	4.1	0.24	0.51

In the **"Results" **section of the **Abstract**, the end of the first sentence should read as follows:

**"**after five years in young adult males (p for trend ≤ .02)."

In the same section, the next sentence should read:

"The positive relationship between maternal encouragement and MVPA approached significance among high-school aged females (p for trend = .06), and a positive relationship between parental encouragement and MVPA approached significance among high school-aged males (p for trend = .05).

In the **"Results" **section of the article, the second sentence should read:

"At Time 2, participants reported engaging in MVPA between 3.9 and 6.8 hours per

week and reported watching TV/video between 17.1 and 19.2 hours per week ..."

In the ***Physical activity ***section of the **Results:**

The end of the second sentence should read:

"years later (p for trend ≤ .02)."

The middle of the third sentence should read:

" engaged in MVPA 2.2 more hours..."

The middle of the fourth sentence should read:

"participating in 5.1 hours of MVPA per week compared to 7.3 hours per week..."

The sixth sentence should read:

"among younger and older males (p for trend ≤ .01)"

and... "among older females (p for trend = .05)" should not be present.

The end of the seventh sentence should read:

"among older males remained (p for trend ≤ 02)"

The end of the eighth sentence should read:

"approached significance (p for trend = .05)."
